# A Facile Preparation of Multicolor Carbon Dots

**DOI:** 10.1186/s11671-022-03661-z

**Published:** 2022-03-08

**Authors:** Risheng Yu, Sen Liang, Yi Ru, Lu Li, Zhikun Wang, Junlang Chen, Liang Chen

**Affiliations:** Department of Optical Engineering, Zhejiang Prov Key Lab Carbon Cycling Forest Ecosy, College of Environmental and Resource Sciences, Zhejiang Provincial Key Laboratory of Chemical Utilization of Forestry Biomass, Zhejiang A&F University, Hangzhou, 311300 China

**Keywords:** Multicolor carbon dots, Solvothermal reaction, Tunable photoluminescence

## Abstract

Carbon dots (CDs) have raised broad interest because of their great potential in the fluorescence related fields, such as photocatalysis and bioimaging. CDs exhibit different optical properties when dissolved in various solvents. However, the effects of solvents during the process of preparation on the fluorescence emission of CDs are still unclear. In this work, CDs were prepared by a simple one-pot solvothermal route. Typical critic acid and thiourea were used as precursors. Through changing the volume ratio of water to *N*,*N*-dimethylformamide (DMF), we have obtained color tunable CDs, with the emission wavelength from 450 to 640 nm. TEM images, Raman and XPS spectra indicate that the particle size of CDs and the content of surface functional groups (C–N/C–S and C≡N bonds) increase with the increasing ratio of DMF to water, which results in the optimal emission wavelength red-shifted. The prepared multicolor CDs may have prospects in the lighting applications.

**Graphical abstract:**

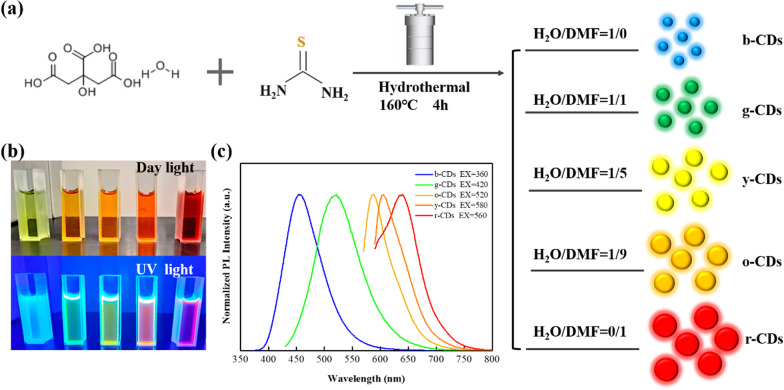

**Supplementary Information:**

The online version contains supplementary material available at 10.1186/s11671-022-03661-z.

## Introduction

Carbon dots (CDs) have attracted extensive interest in the past decades, due to their distinct characteristics, such as abundant raw materials [1], easy to prepare and low toxicity [2]. CDs also own excellent luminescent properties, including excitation and emission wavelength tunable [3, 4]. These unique features endow CDs with the great potential in the optical and biological applications, such as light-emitting devices [5], photocatalysis [6], biosensing [7] and bioimaging [8]. To date, it is still difficult to prepare multcolor CDs, and researchers have put much effort to enlarge the emission spectrum across the entire visible light. For example, Miao et al. have synthesized a kind of CDs with multiple color emission through controlling the extent of graphitization and surface functionalization [9]. With the increasing ratio of critic acid to urea and increasing reaction temperature, the emission wavelengths are shifted from blue to red, due to the increasing conjugation length and the quantity of surface functional groups. Zhu et al. prepared multi-fluorescence CDs via magnetic hyperthermia method in the three different cations [10]. Wang et al. obtained multicolor emitting N-doped CDs under hydrothermal reaction from ascorbic acid and phenylenediamine precursors [11]. Besides, reported CDs can also have multicolor luminescence owing to changing the concentration of the precursors and pH in terms of a constant chemical structure [12].

On the other hand, solvents may play an important role in the photoluminescence (PL) of CDs. For example, Wu’s group has developed a type of CDs with tunable luminescence independent of the excitation wavelength when dispersed in different solvents [13]. Similarly, Mei et al. have obtained amphipathic CDs with tunable emission from blue to green and excitation-independent property when dissolved in different solvents [14]. Ding et al. successfully prepared CDs with wide range wavelength by changing the solvent in reactions and found that the solvent controlled the carbonization processes during the solvothermal reactions [15]. Affecting tunable optical property is ascribed to the interactions between the surface groups of CDs and solvent molecules, including hydrogen bonding [16] and dipole–dipole interactions [17]. Bai et al. have synthesized multicolor CDs through solvent-responded strategy using r-CDs as initiator. Solvent adhesion or various emissive defects on the surface of CDs can produce tunable luminescence in the various solvents [18]. Tian et al. obtained multicolor CDs through controlling bandgaps emission in three different solvents. The extents of decomposition and carbonization of precursors lead to the emission wavelength shift from blue to red of CDs [19]. Wang’s group found that CDs could emit excitation-independent fluorescence from green to red when the as-prepared CDs dispersed in the different solvents, which is attributed to the intramolecular charge transfer [20]. Wei et al. have shown tunable emission luminescence when the as-synthesised NCDs dispersed in different solvents [21].

The above studies are focused on the effects of solvent in the post-treatment of CDs. However, the effects of solvents during the process of preparation on the fluorescence emission of CDs are still unclear. In this work, we prepared a kind of CDs, using a simple one-pot solvothermal route. Critic acid and thiourea were adopted as precursors. Water and *N,N*-dimethylformamide (DMF) were used as solvents. We mainly investigated the influence of solvents through changing the volume ratio of water to DMF. The obtained CDs are color tunable, with the emission wavelength from blue to red. In addition, TEM images, Raman and XPS spectra were employed to characterize the particle size of CDs and the content of surface functional groups.

## Materials and Methods

### Materials

Hydrated critic acidic (C_6_H_10_O_8_), thiourea (CH_4_N_2_S), DMF (C_3_H_7_NO), ethyl acetate (C_4_H_8_O_2_), petroleum ether (30–60℃) were used in the preparation of CDs. All these agents were purchased from Shanghai Aladdin Biochemical Technology Co. Ltd. Deionized water was used with 18.2 MΩ cm^−1^ in all experiments.

### Synthesis of Multicolor CDs

**Fig. 1:**
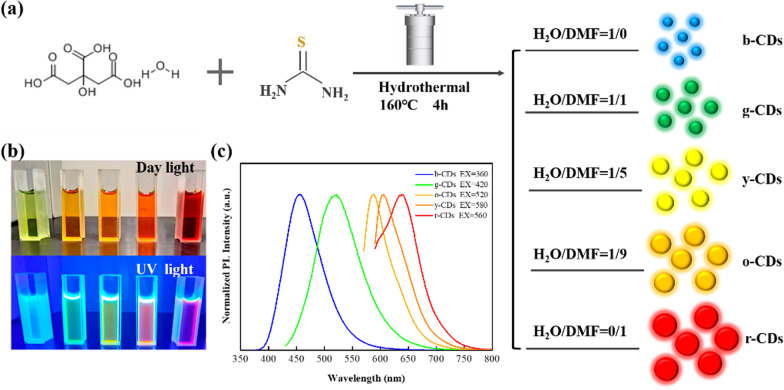
**a** One-pot hydrothermal synthesis route for CDs with distinct fluorescence characteristics. **b** Five CD samples under day light and 365 nm UV light. **c** Corresponding PL emission spectra of the five samples, with maximums at 440 nm, 530 nm, 580 nm, 610 nm, and 640 nm, respectively

Multicolor CDs were prepared by a one-spot solvothermal route (Fig. 1a) using a series of volume ratios of water to DMF. In detail, 1.26 g (0.2 mol/L) hydrated critic acidic and 1.37 g (0.6 mol/L) thiourea were dissolved in 30 ml mixed solution with various volume ratios of H_2_O to DMF, namely, 1:0 (pure water), 1:1, 1:5, 1:9 and 0:1 (pure DMF). Then, each solution was translated into a Teflon-lined stainless-steel autoclave, followed by heating at 160 ℃ for 4 h. The corresponding prepared CDs were denoted as b-CDs, g-CDs, y-CDs, o-CDs, and r-CDs (Fig. 1b), respectively. After that, these solutions were filtrated by 0.22 μm membranes. Then, the purified solutions were added into the mixed solvent of petroleum ether and ethyl acetate to remove redundant DMF. Finally, the obtained CDs were used in the following characterizations.

### Characterizations

The absorbance of multicolor CDs was detected by a Shimadzu UV-25500 PC UV/Vis absorption spectrometer. The functional groups of CDs were measured by Fourier transform infrared spectroscopy (FT-IR, Thermo Scientific Nicolet iS50, America) over the range of 800–4000 cm^−1^. All fluorescence spectra were performed by a fluorescence spectrophotometer (F-4600, Hong Kong Tian Mei Co., Ltd.). Raman spectra were measured by a HORIBA Scientific LabRAM HR Evolution high resolution Raman spectrometer with laser frequency of 785 nm as an excitation source. The X-ray photoelectron spectroscopy (XPS) experiments were performed by the ThermoFisher ESCALAB 250Xi spectrometer. The Atomic Force Microscopy (AFM, Bruker, Multimode-8) was employed to obtain the heights and sizes of CDs. The X-ray diffraction (XRD) characterization of CDs was conducted by the Rigaku Ultima IV. High-resolution transmission electron microscopy (HRTEM) micrographs were acquired at room temperature by the FEI F200C TEM operating at 200-kV.

## Results and Discussion

### Characterizations of b-, g- and r-CDs

**Fig. 2:**
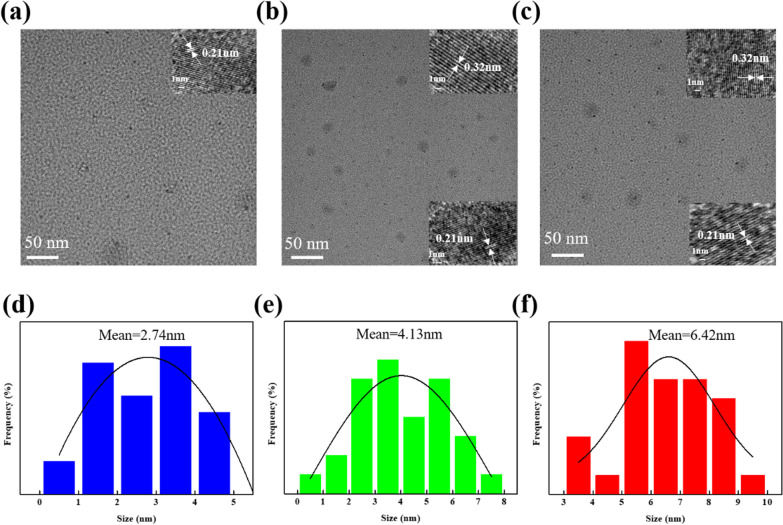
TEM images of b-CDs (**a**), g-CDs (**b**) and r-CDs (**c**). Insets specify the HRTEM images. The histograms of size distribution of b-CDs (**d**), g-CDs (**e**) and r-CDs (**f**)

Because the fluorescence of CDs is related to the particle size, the type and content of the functional groups, we performed a series of characterizations, taking b-CDs, g-CDs and r-CDs as examples. The size and morphology of CDs are explored by TEM, as shown Fig. 2a–c. Based on the histogram of size distribution, the average sizes of b-CDs, g-CDs and r-CDs are 2 ± 1 nm, 4 ± 1 nm and 6 ± 1 nm, respectively (Fig. 2d–f). In addition, the HRTEM images highlight that the b-CDs have a crystalline lattice fringe of 0.21 nm, corresponding to the lattice plane (100) of graphic carbon [22]. The g-CDs and r-CDs possess crystalline lattice fringes of 0.21 nm and 0.32 nm, corresponding to the lattice planes (100) and (002) of graphic carbon [23]. The XRD pattern of b-CDs exhibits a narrow peak centered at 6.8 Å (see Additional file 1: Figure S1a). The XRD patterns of g-CDs and r-CDs show not only a narrow peak located at 6.8 Å, but a broad peak center at 3.4 Å. The XRD results indicate that the b-CDs, g-CDs and r-CDs consist of small crystalline cores with a disordered surface, similar to the graphite lattice spacing [14, 24]. The AFM images present the height distribution of b-CDs, g-CDs and r-CDs (see Additional file 1: Figure S1(b–d)). The average height of b-CDs, g-CDs and r-CDs is approximate 3 nm. These results clearly show that the particle sizes of CDs become larger gradually from b-CDs to r-CDs.

**Fig. 3:**
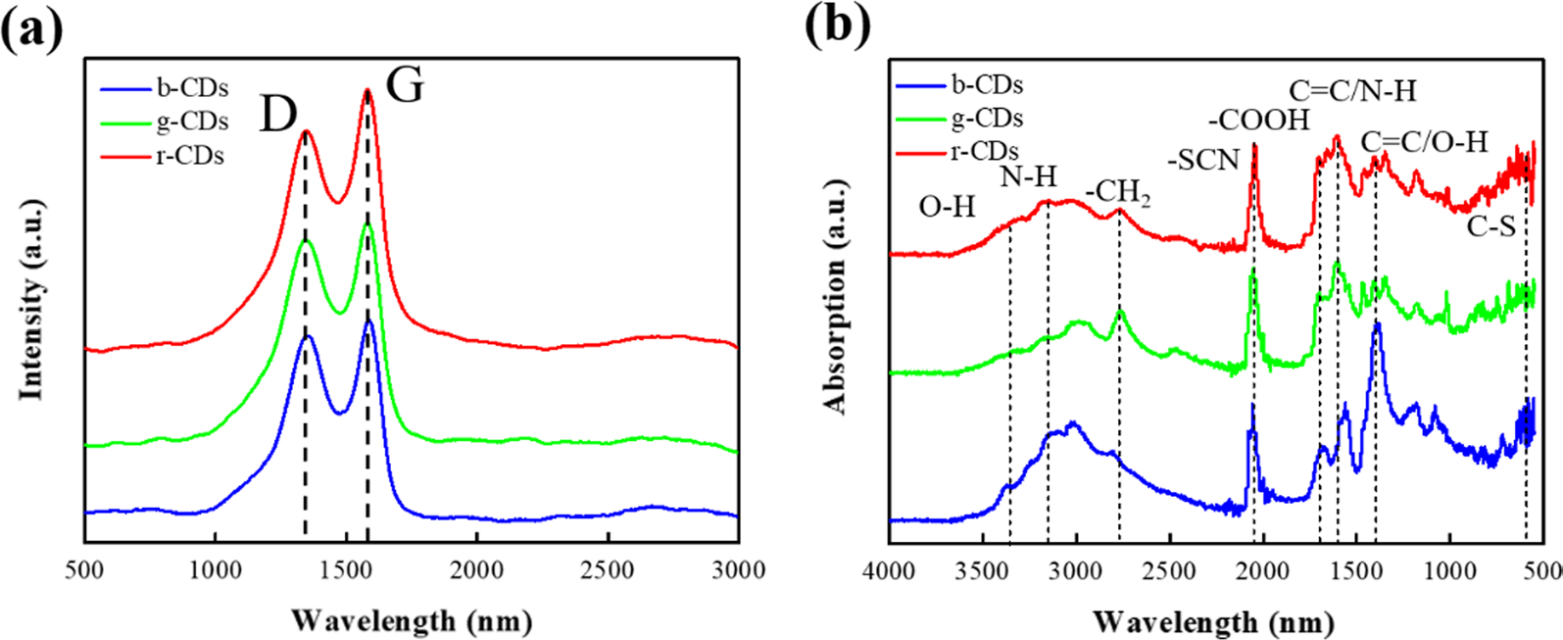
Raman spectroscopy (**a**) and FT-TR spectroscopy (**b**) of the three selected CDs

Figure 3a shows the Raman spectra of b-CDs, g-CDs and r-CDs, in which a G band at 1573 cm^−1^ and a D band at 1342 cm^−1^ correspond to the graphitic sp^2^ carbon structures and disordered sp^3^ carbon structures [25]. The ratios of I_G_/I_D_ are 1.11, 1.20 and 1.24 for b-CDs, g-CDs and r-CDs, implying the higher graphitization degree of CDs with the increasing ratio of DMF to water, consistent with the TEM and AFM results. FT-IR and XPS characterizations were further performed to investigate the type and content of the functional groups on the b-CDs, g-CDs and r-CDs. The FT-IR spectra of CDs are shown in Fig. 3b. The emerging peaks at 570–600 cm^−1^ (C–S bonding) [26] and 2050 cm^−1^ (–SCN bonding) [27] reveal the nitrogen and sulfur doping in the CDs. The peaks at ~ 3370 cm^−1^ and 3160 cm^−1^ are stretching vibrations of O–H [28] and N–H. The peaks at 1710 cm^−1^, 1610 cm^−1^ and 1410 cm^−1^ are designated to the ν_C=O_ of the -COOH groups, the bending vibration of C = C/N–H and C = C/O–H [27], respectively. Obviously, the order of the content of the oxygen-containing groups (especially for O–H) is b-CDs > g-CDs > r-CDs, which is opposite to the order of the particle size. These results demonstrate that the volume ratio of DMF to water in the solvothermal reaction has a significant effect on the particle size and the functional groups of CDs.

**Fig. 4:**
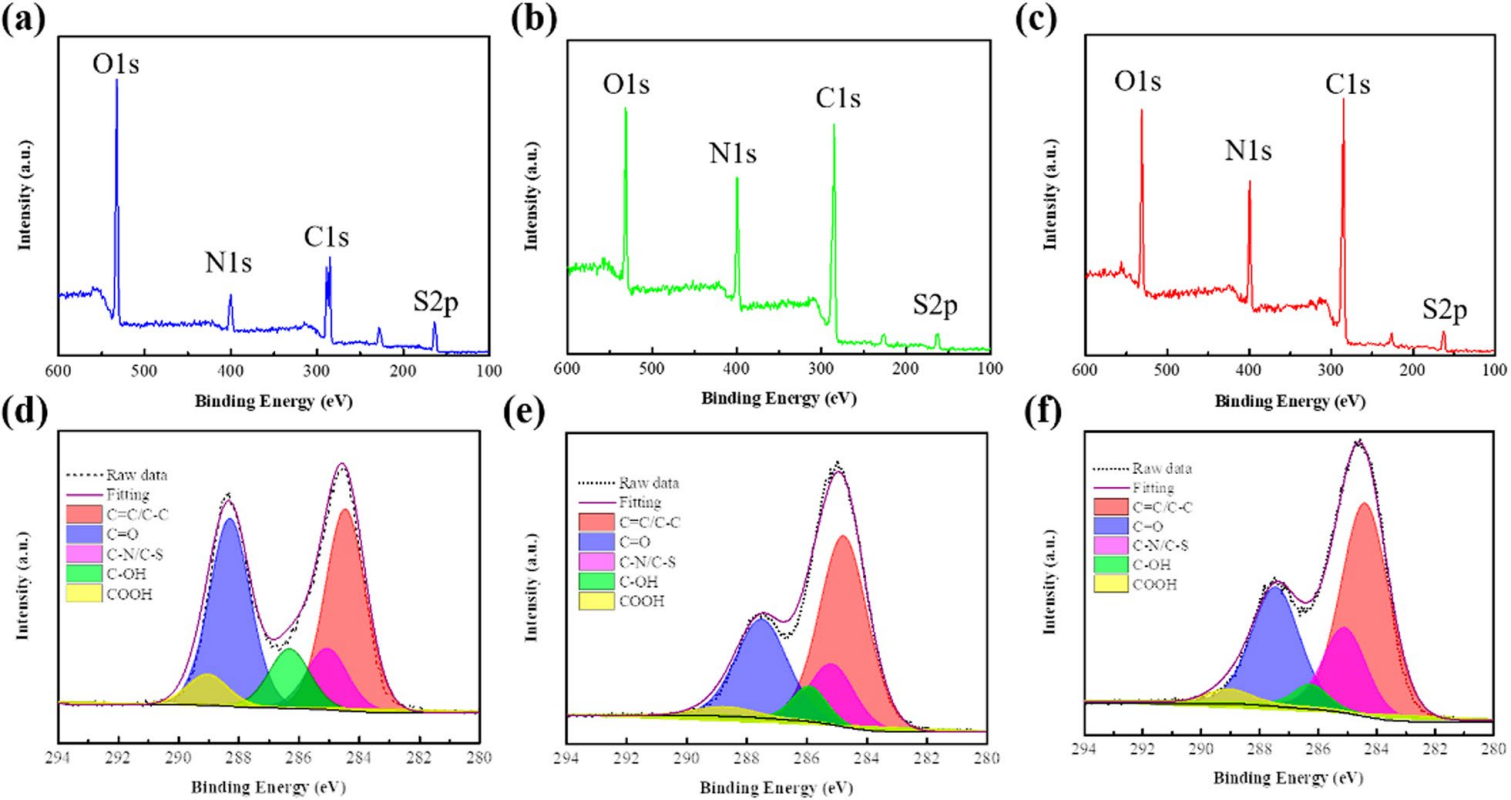
XPS full-scan survey analysis of **a** b-CDs, **b** g-CDs, **c** r-CDs and C1s high-resolution level spectra of **d** b-CDs, **e** g-CDs and **f** r-CDs

Furthermore, the atomic contents and functional groups of b-CDs, g-CDs and r-CDs were characterized by XPS. As shown in Fig. 4a–c, the four diagnostic peaks located at 531 eV, 400 eV, 285 eV, and 163 eV correspond to O1s, N1s, C1s, and S2p, respectively. The ratios of O/C were 75%, 25% and 24% for b-CDs, g-CDs and r-CDs. The C1s spectra are divided into five peaks, namely, C=C/C–C (284.5 eV), C–N/C–S (285.1 eV), C–OH (286.3 eV), C=O (288.3 eV), and O=C–OH (289.0 eV) [26] (Fig. 4d–f). The sequence of the content of the oxygen-containing groups is b-CDs > g-CDs > r-CDs, consistent with the FT-IR results. In addition, the high-resolution N1s XPS spectra of b-CDs, g-CDs, and r-CDs are fitted by three components centered at C≡N (397.4 eV), pyrrolic N (399.4 eV), and graphite N (401.2 eV), respectively (see Additional file 1: Figure S2(a–c)). The high resolution spectra of the S2p also clearly show the peaks at 164.5 eV and 165.9 eV, corresponding to S2p^3/2^ and S2p^3/1^ spectra of the C–S–C bond in thiophene-type structure due to the spin–orbit splitting [29], which is agreement with sulfone bridges(–C–SO_X_–C) [29] (Figure S2d–f). From the detailed analyses of the N1s and S2p spectra in Table S1, the contents of C–N/C–S and C≡N bonds increase with the increasing ratio of DMF to water, compared with the decrease of the contents of oxygen-containing groups.

### Optical Properties of b-, g- and r- CDs

**Fig. 5:**
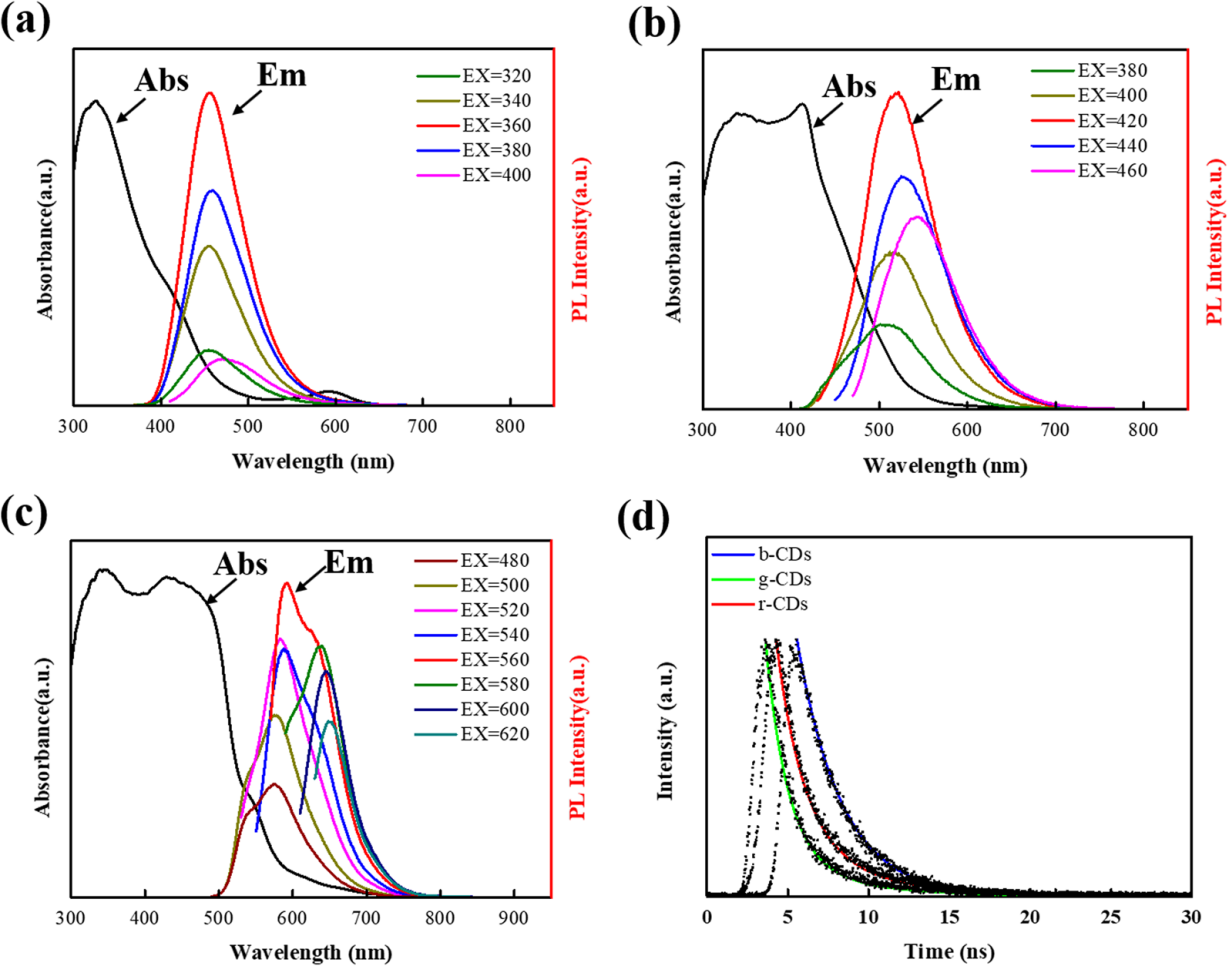
UV/Vis and PL emission spectra of b-CDs (**a**), g-CDs (**b**) and r-CDs (**c**). **d** PL decay curves of b-CDs, g-CDs and r-CDs

The UV–Vis absorption spectra of CDs present a well resolved *n*–π* transition at 320 nm [30], which originates from the functional groups of C=X (X=N, S, O). Meanwhile, b-CDs, g-CDs and r-CDs exhibit energy absorption bands at about 360 nm, 420 nm and 560 nm, respectively, as shown in Fig. 5a–c. Such energy bands are classically associated with the narrowing of electronic bandgaps, which leads to the fluorescence red-shift [31]. The position of energy absorption bands demonstrates the wavelength region of fluorescent excitation. The optimal emission wavelengths of b-CDs and g-CDs are 440 nm and 530 nm, respectively. Unlike b-CDs and g-CDs, there are dual-emissive wavelengths located at 600 nm and 640 nm for r-CDs. With the increasing excitation wavelength, the PL peaks exhibit slight fluctuations, which elucidates the excitation-dependent properties of b-CDs, g-CDs and r-CDs, implying a possible carbogenic core state emission [32]. Under solvothermal conditions, decomposition performed between critic acid and thiourea to form N, S-doped CDs with abundant-SCN, -NH_2_ on their surface. Obviously, the volume ratio of water to DMF can affect the extents of decomposition and carbonization in the reaction process [29]. It can be speculated that the decomposition of precursors and carbonization of solvents gradually increase with the higher volume ratio of DMF to water, resulting in red-shifted absorption and emission bands, which is well consistent with their increased particle sizes and functional groups of CDs. In addition, the PL decay curves (Fig. 5d) of b-CDs, g-CDs and r-CDs are fitted by the dual-exponential curves. The results show that the lifetimes of b-CDs, g-CDs and r-CDs are 2.75 ns, 4.67 ns and 4.88 ns, respectively.

### Effects of Reaction Conditions and Solvents on PL Properties

**Fig. 6:**
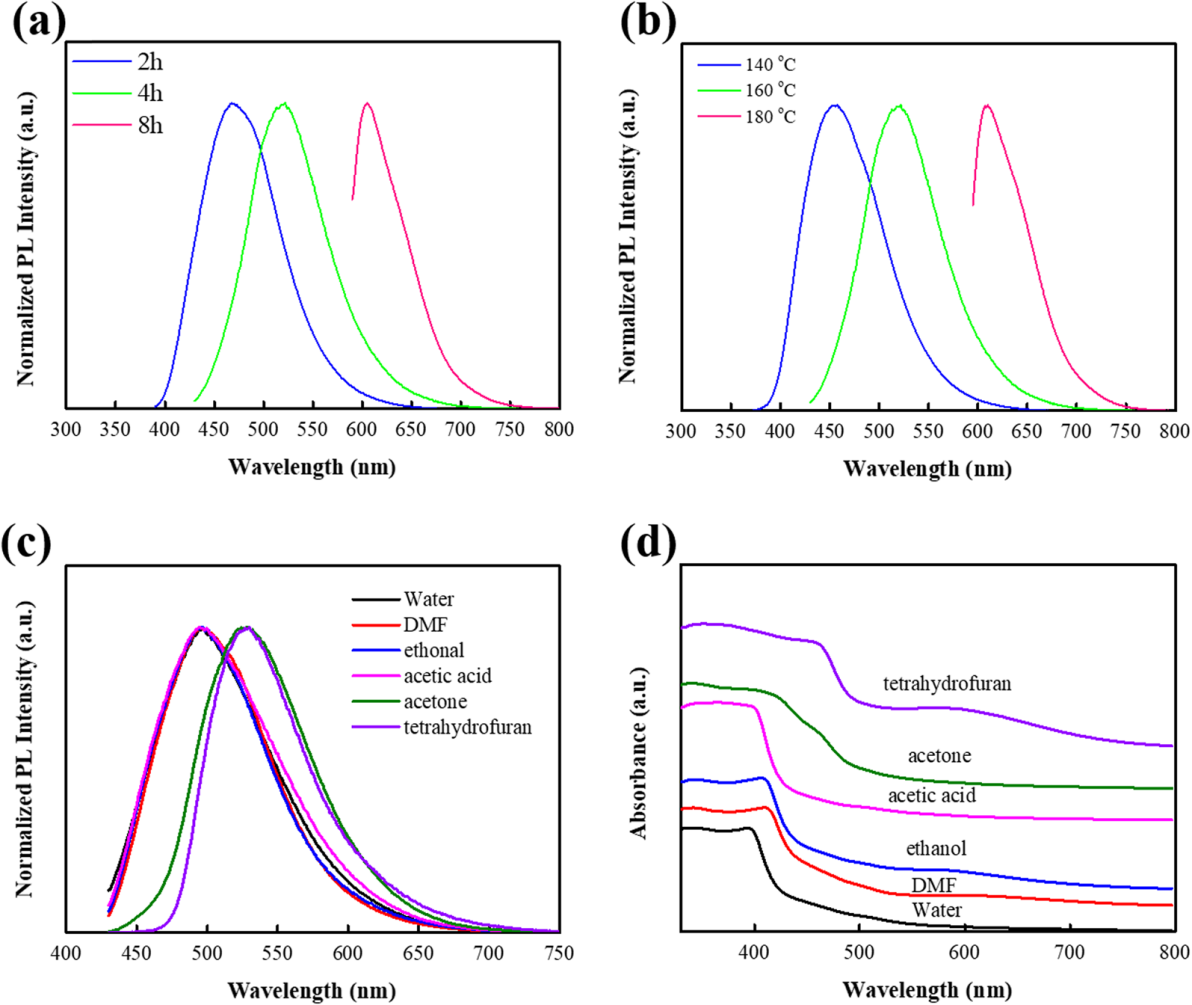
Normalized PL emission spectra of g-CDs with **a** different heating times, **b** different heating temperature. **c** Normalized PL spectra (*λ*_ex_ = 420 nm) and **d** UV–vis absorption spectra of g-CDs dispersed in six solvents

Furthermore, we investigated the effects of reaction conditions (reaction time and temperature) on the preparation of multicolor CDs, taking g-CDs as an example. As shown in Fig. 6a, b, the maximum emission wavelengths have dramatically red-shifted with the reaction time prolonged from 2 to 8 h, and the reaction temperature increased from 140 to 180℃. When the heating time increases from 2 to 8 h at 160 ℃, the maximum emission peaks increase from blue (460 nm) to red (605 nm). Similarly, when the reaction temperature from 140 to 180 ℃ at 4 h, the maximum emission peaks increase from blue (450 nm) to red (610 nm). This phenomenon indicates that the increase of reaction time and reaction temperature leads to the PL red-shift of CDs, which is ascribed to the carbonization in the materials [9]. It has been reported that longer thermal time and higher temperature will promote the carbonization of precursors [9]. Based on these results, we can speculate that the emissive wavelength of CDs strongly depends on the carbonization degree of precursors, which is in line with the particle size and functional groups on CDs.

To explore the effects of solvents on CDs, we have performed additional experiments of g-CDs dispersed in six different solvents, namely, water, DMF, ethanol, acetic acid, acetone, and tetrahydrofuran (THF), which are in the sequence of polarity from strong to weak. In stronger polar solvents, the PL emission wavelength of g-CDs is related to the excitation wavelength (see Additional file 1: Figure S3(a–d)). On the contrary, the PL emission wavelength is independent on the excitation wavelength in weaker polar solvents (see Additional file 1: Fig. S3(e–f)). Figure 6c demonstrates that the PL emission wavelength of g-CDs red shifts in weaker polar solvents compared with in stronger polar solvents. This is because weak polar solvents will affect the electronic structure and then reduce the energy gap of g-CDs [33]. UV–vis absorption spectra (Fig. 6d) show that absorption wavelength red shifts of g-CDs in weak polar solvents, which further confirms that weak polar solvents play an important role in affecting *n*–π* transition, leading to the emission spectra red-shift [34, 35].

## Conclusions

In summary, we have developed a facile and feasible way to synthesis multicolor CDs, which fluorescence covers a majority of the visible spectrum. Through adjusting the volume ratio of water to DMF, the obtained CDs are color tunable, with the emission wavelength from blue to red. We find that solvent (DMF) plays an important role in preparing multicolor CDs, because DMF is decomposed in the carbonization process. With the increasing ratio of DMF to water, the particle sizes of CDs become larger gradually, and more functional groups are formed on the surface of CDs, which lead to the PL red-shift of CDs. Our method can enlarge the visible spectrum of CDs and the prepared multicolor CDs may have application prospects in the optical and biological fields of light-emitting devices and bioimaging systems.

## Supplementary Information

**Additional file 1**. XRD and AFM characterizations of b-CDs, g-CDs and r-CDs; XPS Data analysis of b-CDs, g-CDs and r-CDs; Solvents effect PL emission spectra of g-CDs.

## Data Availability

The datasets used or analysed during the current study are available from the corresponding author on reasonable request.

## Abbreviations

CDs: Carbon dots
PL: Photoluminescence
XRD: X-ray diffraction
HRTEM: High-resolution transmission electron microscopy
FT-IR: Fourier transform infrared
XPS: X-ray photoelectron spectroscopy
AFM: Atomic Force Microscopy
DMF: *N*,*N*-Dimethylformamide
UV/Vis: Ultraviolet and Visible spectrophotometry
H_2_O: Deionized water
