# Supplementary material for: A Facile Preparation of Multicolor Carbon Dots
Source: Nanoscale Res Lett. 2022 Mar 8;17:32. doi: 10.1186/s11671-022-03661-z (PMC8904681; doi:10.1186/s11671-022-03661-z)
Supplement: Supplementary file 1 — Additional file 1. XRD and AFM characterizations of b-CDs, g-CDs and r-CDs; XPS Data analysis of b-CDs, g-CDs and r-CDs; Solvents effect PL emission spectra of g-CDs. [file 11671_2022_3661_MOESM1_ESM.docx]

**Supporting information to**

**A facile preparation of multicolor carbon dots**

**by**

Risheng Yu, Sen Liang, Yi Ru, Zhikun Wang, Junlang Chen*, Liang Chen*

*Department of Optical Engineering, Zhejiang Prov Key Lab Carbon Cycling Forest Ecosy, College of Environmental and Resource Sciences, Zhejiang Provincial Key Laboratory of Chemical Utilization of Forestry Biomass, Zhejiang A&F University, Hangzhou 311300, China*

*Corresponding author. E-mail: [chenjunlang7955@sina.com](mailto:chenjunlang7955@sina.com) (J.C.),

liangchen@zafu.edu.cn (L.C.).

**Contents**

[PS1 XRD and AFM characterizations 2](#_Toc87264628)

[PS2 XPS Data analysis 3](#_Toc87264629)

[PS3 Solvents effect PL emission spectra of g-CDs 4](#_Toc87264630)

# PS1 XRD and AFM characterizations


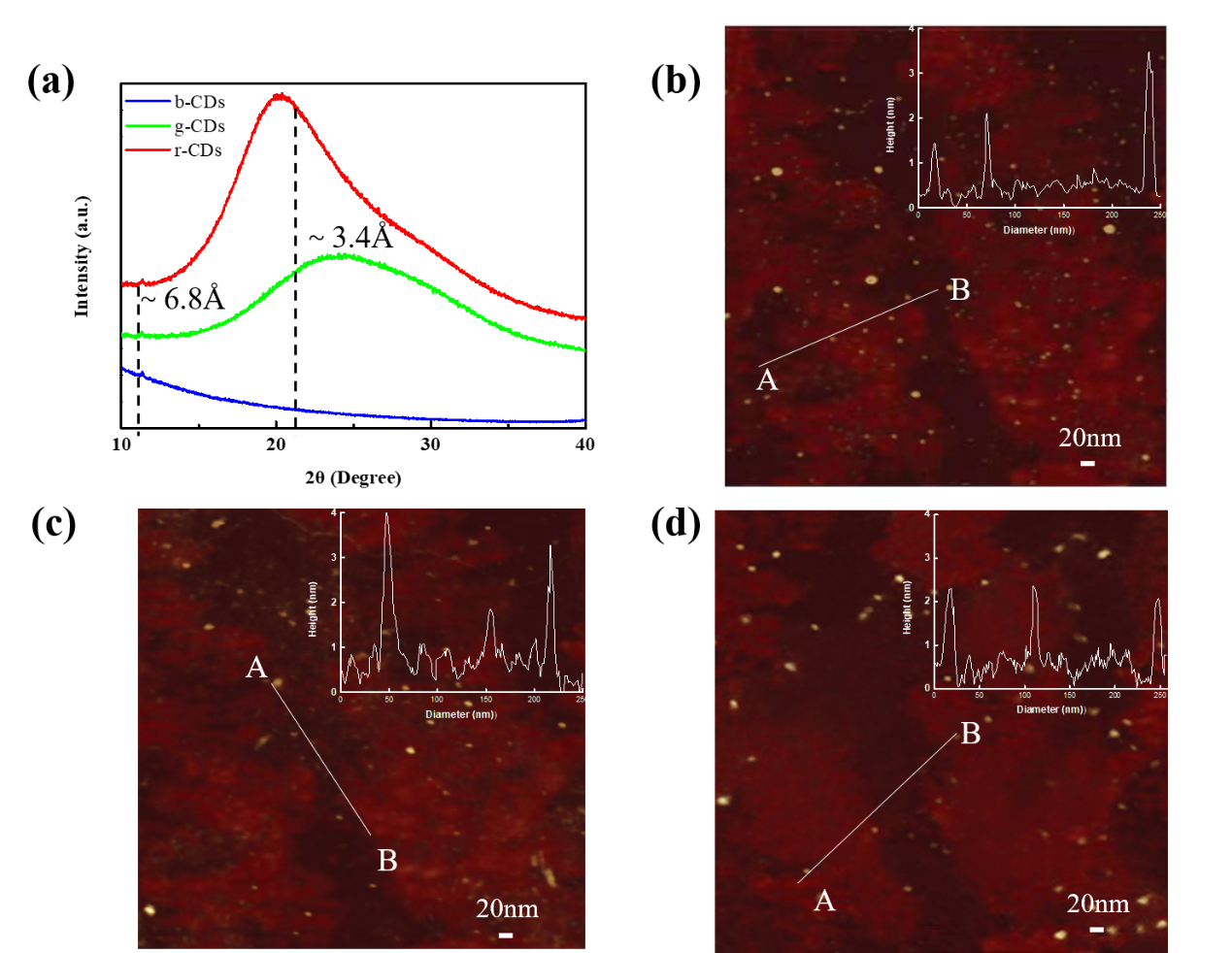


**Figure S1.** The XRD patterns **(a)** of b-CDs, g-CDs and r-CDs. AFM images the corresponding height **(b)** of b-CDs, **(c)** of g-CDs, **(d)** of r-CDs

# PS2 XPS Data analysis


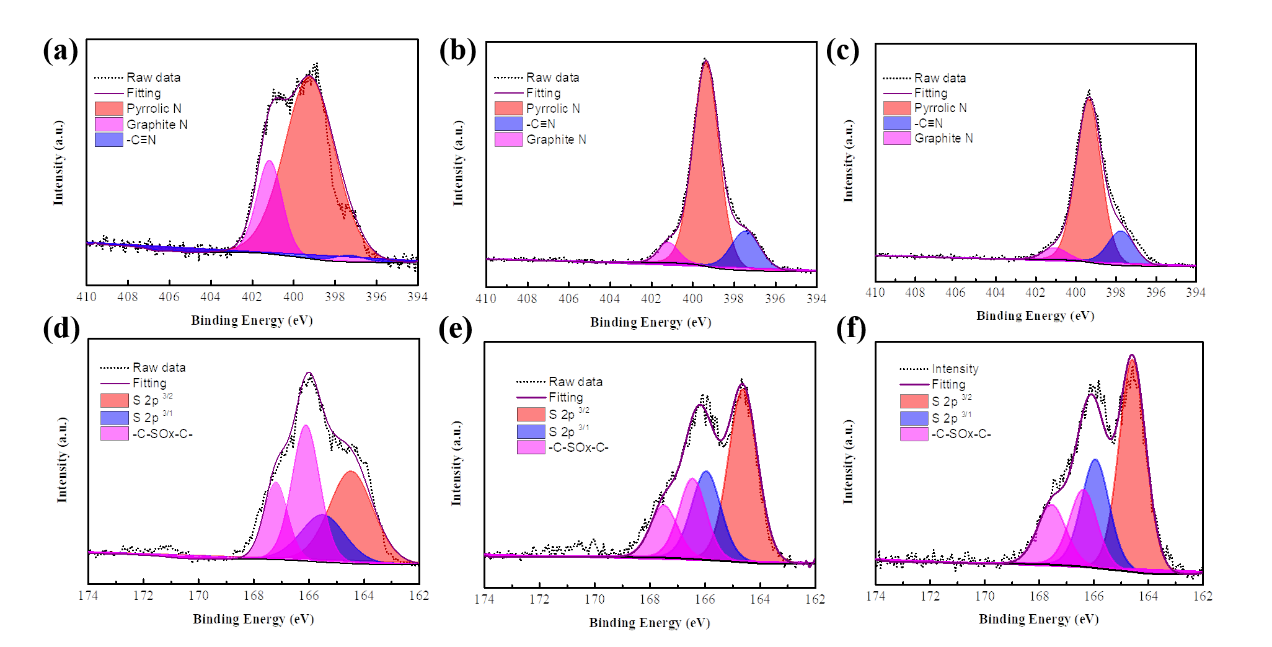


**Figure S2.** N1s high-revolution level spectra **(a)** of b-CDs, **(b)** of g-CDs, **(c)** of r-CDs and S2p high-revolution level spectra **(d)** of b-CDs, **(e)** of g-CDs, **(f)** of r-CDs.

**Table S1.** XPS Data Analyses of the C1s, N1s and S2p Spectra of three typical CD Samples.

|  | C=C/C-C (%) | C=O (%) | C-N/C-S (%) | C-OH (%) | COOH (%) |
| --- | --- | --- | --- | --- | --- |
| b-CDs | 37.09 | 35.04 | 11.26 | 10.97 | 5.59 |
| g-CDs | 49.24 | 26.93 | 14.34 | 6.38 | 2.70 |
| r-CDs | 49.11 | 27.28 | 17.34 | 3.64 | 2.63 |

|  | Pyrrolic N (%) | Graphite N (%) | -C≡N (%) |
| --- | --- | --- | --- |
| b-CDs | 78.43 | 20.15 | 1.42 |
| g-CDs | 77.88 | 5.88 | 16.24 |
| r-CDs | 77.70 | 5.20 | 17.10 |

|  | S 2p 3/2    S 2p 3/1  (%) | -C-SOx-C- (%) |
| --- | --- | --- |
| b-CDs | 36.54 | 63.48 |
| g-CDs | 51.93 | 48.07 |
| r-CDs | 54.18 | 45.82 |

# PS3 Solvents effect PL emission spectra of g-CDs


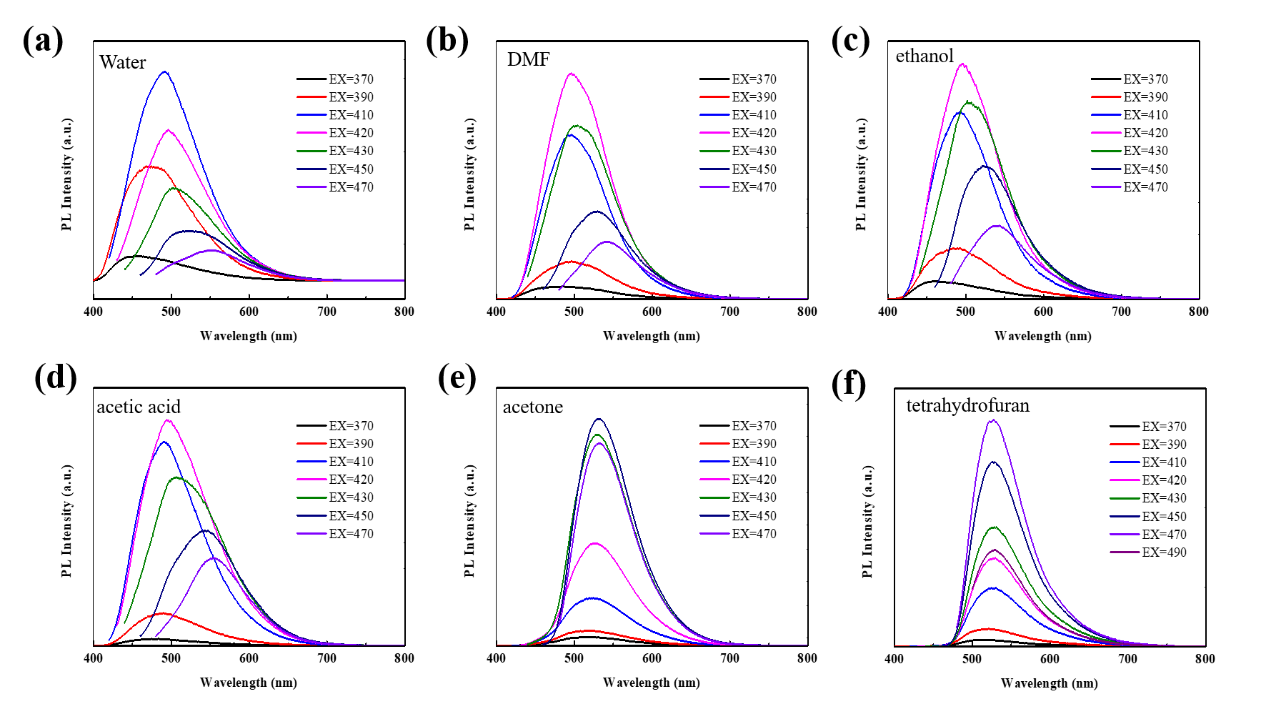


**Figure S3.** PL spectra of g-CDs dispersed in the solvents of Water **(a)**, DMF**(b)**, ethanol **(c)**, acetic acid **(d)**, acetone **(e)**, and tetrahydrofuran **(f)** excited by different excitation wavelength.
